# Influence of hydropower stations on the water microbiota in the downstream of Jinsha River, China

**DOI:** 10.7717/peerj.9500

**Published:** 2020-07-16

**Authors:** Xiaojuan Chen, Da He, Lianfeng Zhou, Yankun Cao, Zhanjing Li

**Affiliations:** 1 Key Laboratory of Ministry of Water Resources for Ecological Impacts of Hydraulic-Projects and Restoration of Aquatic Ecosystem, Institute of Hydroecology, Ministry of Water Resources & Chinese Academy of Sciences, Wuhan, China; 2 Research and Development Center, Guangdong Meilikang Bio-Science Ltd., Dongguan, China

**Keywords:** Aquatic bacterial community, Hydropower station, Jinsha River, Microbiota

## Abstract

Construction of hydropower stations has been an important approach to meet China’s increasing power demand, but the impact of construction of hydropower stations on river microbiota is not fully understood. To evaluate this, the microbial composition from 18 sampling sites in the downstream of Jinsha River of China, upstream and downstream of two completed and two under-construction hydropower stations, were analyzed using high-throughput 16S rRNA gene sequencing. Three independent samples from each site were analyzed. A total of 18,683 OTUs from 1,350 genera were identified at 97% sequence similarity. Our results showed that the completion of hydropower stations would significantly increase the relative abundances of Acidobacteria, Chlorobi, Chloroflexi, Cyanobacteria, Nitrospirae, and Planctomycetes, especially the relative abundance of *Synechococcus* dOTUs and thus increase the risk of algal blooms. PCA based on all KEGG pathways and the significantly different KEGG pathways showed the predicted metabolic characteristics of the water microbiota by PICRUSt in the activated hydropower station group were significant difference to the other groups. Results from canonical correspondence analysis showed that water temperature and dissolved oxygen had significant effects on microbiota composition. These results are important for assessing the impact of hydropower stations on river microbiota and their potential environmental risks.

## Introduction

**Figure 1:**
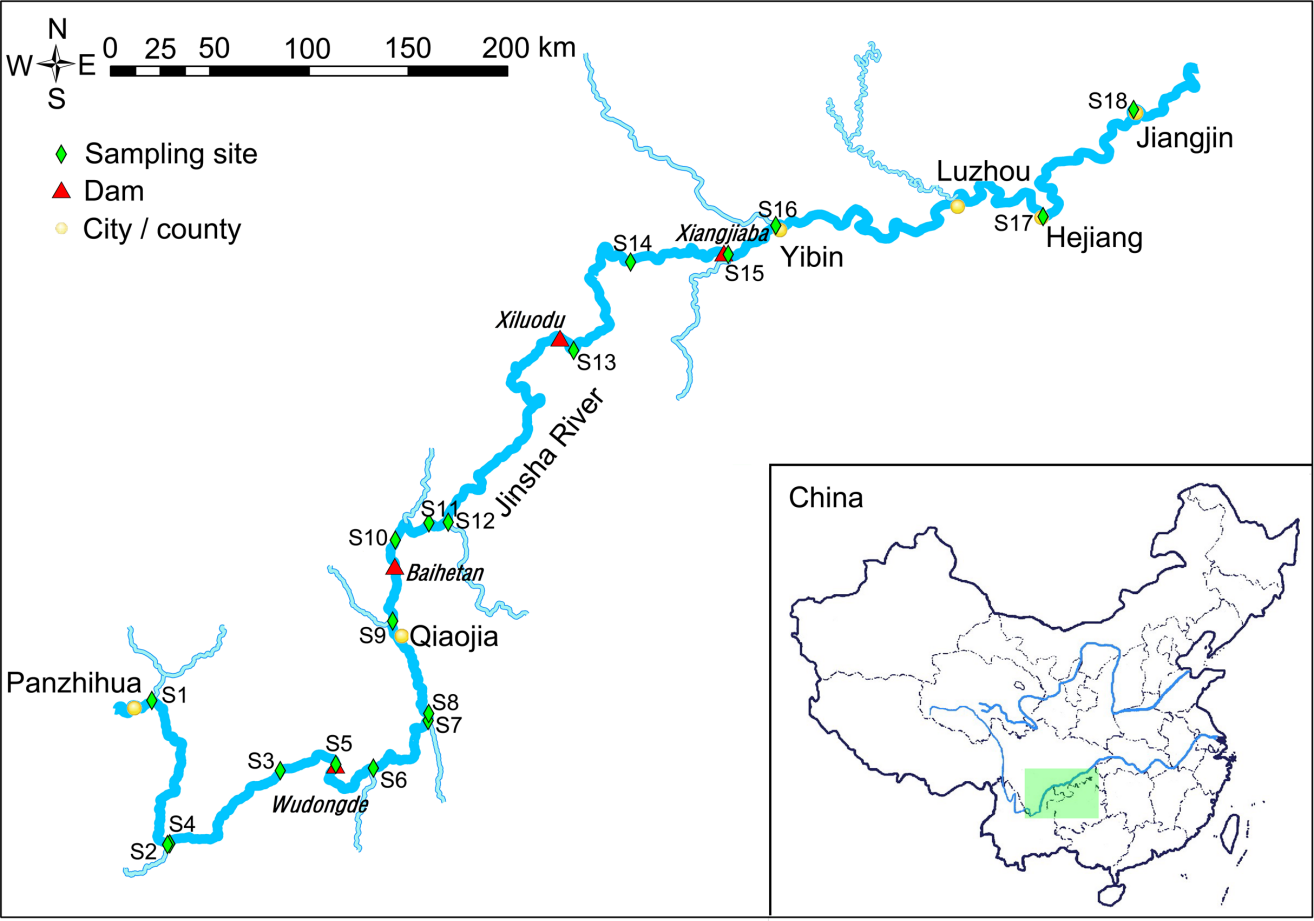
Map of the sampling sites. The light green rectangle shows the sampling area locating in the upstream of the Yangtze River, China.

With changes in the global fossil energy supply and global climate, the development of green energy sources such as hydropower and wind energy has become an important alternative for China (Huang & Yan, 2009; Chang, Liu & Zhou, 2010; Wang et al., 2013). The Jinsha River is upstream of the Yangtze River and is rich in hydropower resources. The hydropower reserve of this stretch of river is 1.12 × 10^8^ kW, which is the highest of the thirteen planned hydropower bases in China (Fan, 2017). The current plan is to build four hydropower stations in the downstream of Jinsha River that is, Xiangjiaba, Xiluodu, Baihetan, and Wudongde from downstream to upstream (Fig. 1; Chen, Deng & Liang, 2010; Chen et al., 2014; Fan, 2017). The total installed capacity of the four cascade hydropower stations is 4,4800 mw, with an average annual power generation of 191.22 billion kW·H. The Xiangjiaba and Xiluodu hydropower stations were put into operation in July 2014. The Baihetan and Wudongde hydropower stations are currently under construction, and are expected to be completed in 2021 and 2020, respectively (Li, Fan & Cheng, 2006; Yin et al., 2015).

Construction of hydropower stations results in changes to the hydrology, water environment and aquatic habits, which result in significant changes to the structure and function of the original river ecosystem (Friedl & Wüest, 2002; Chen, Deng & Liang, 2010). For instance, the construction of hydropower stations decreases flow rates and increases the water level and residence time in the upstream of the hydropower stations, which probably increases the risk of freshwater harmful algal blooms (FHAB) as the conditions facilitate their occurrence (Paerl & Huisman, 2008; Hudnell et al., 2010; Paerl et al., 2016; Paerl, Otten & Kudela, 2018).

Cyanobacteria are the main bacterioplanktonic species causing the FHAB (Paerl et al., 2016). Bacterioplankton community is an important part of the freshwater ecosystem and plays an important role in nutrition and energy cycles (DeLong, 2004; Jetten, 2008; Ni et al., 2010). Changes to the freshwater bacterioplanktonic community caused by human activities not only affect the health and community structure of other aquatic and terrestrial organisms (Van der Merwe et al., 2012; Hiborn et al., 2014; Qiao et al., 2016), but also change the structure and function of the whole aquatic ecosystem in the long term through biogeochemical processes (Chutter, 1995; Friedl & Wüest, 2002). Therefore, it is of great importance to evaluate the impact of the construction of artificial hydropower stations on the aquatic bacterial community of the river and to assess the risk of FHAB. So far, however, there is few theoretical frameworks in this area.

Considering the influence of hydropower station construction on river hydrology and nutrients, and the important role of bacterioplankton community in the process of nutrient and energy cycles, we hypothesized that construction of hydropower stations results in significant changes of bacterioplankton community in river water, and the changes will increase the risk of FHAB. To test our hypothesis, we analyzed the changes of the bacterioplanktonic community composition in the downstream of the Jinsha River (Fig. 1), and assessed the impact of the cascade of hydropower stations on the composition of the river microbiota in this study.

## Materials and Methods

### Sample collection

The water samples were collected from the downstream of the Jinsha River in May, 2019 (Fig. 1). Sampling sites 1–4 were in the natural river upstream of the Wudongde hydropower station. Sampling sites 5 to 9 were between the Wudongde and the Baihetan hydropower stations. These two hydropower stations were under construction, and therefore the section between them was the natural river. Sample sites 10–12 were between the Baihetan and the Xiluodu hydropower stations. This section of the river had become a reservoir from July 2014. Sampling sites 13 and 14 were between the Xiluodu and the Xiangjiaba hydropower stations. This section of the river had also become a reservoir from July 2014. Sampling sites 15–18 were downstream the Xiangjiaba hydropower station, which is the National Nature Reserve of Rare and Endemic Fish in the upstream section of the Yangtze River (Fig. 1; Fig. S1). The sampling sites were divided into four groups according to the construction of hydropower station and whether there is a tributary at the sampling site, i.e. principal stream group (S3, S4, S8, S11, S14, S17, and S18), tributary into group (S1, S2, S6, S7, S9, S12, and S16), building hydropower station group (S5 and S10), and activated hydropower station group (S13 and S15).

Each sample consisted of approximately 500 mL of water collected 50 cm below the water surface from the river shore filtered through glass-fiber membranes (GF/C) with 0.22 μm apertures to extract microbial DNA as previously described (Ni et al., 2010). Three independent water samples 5 m apart were collected from each sampling site. Then the glass-fiber membranes were store at −20 °C until DNA extraction. At the same time, pH, dissolved oxygen (DO), water temperature (WT), conductivity, total dissolved solids (TDS), and salinity were measured using a YSI 6600 multi parameter water quality monitor (YSI, USA). The transparency of the water was determined as described in a previous report (Huang, 2000).

### DNA extraction and high-throughput sequencing

The filtrating glass-fiber membranes were cut into pieces respectively and microbial DNA was extracted using a PowerSoil DNA isolation kit (Mo Bio Laboratories, Inc., Carlsbad, CA, USA). DNA concentration and quality were checked using a NanoDrop spectrophotometer (Thermo Fisher Scientific, Waltham, MA, USA).

The V4–V5 hypervariable region of the prokaryotic 16S rRNA gene was amplified using the universal primer pair 515F and 909R, with a 12-nt sample-specific barcode sequence included at the 5′-end of the 515F sequence to distinguish between samples (Huang et al., 2018; Ni et al., 2019). Polymerase chain reaction was performed and the amplicons were sequenced using a HiSeq system at Guangdong Meilikang Bio-Science, Ltd. (Guangdong, China), as described previously (Ni et al., 2017; Huang et al., 2018; Xiang et al., 2018).

The raw sequences were merged using FLASH-1.2.8 software (Magoc & Salzberg, 2011) and processed using the QIIME pipeline 1.9.0 with default parameters (Caporaso et al., 2010). Chimeric sequences were identified and removed using the Uchime v4.2.40 algorithm before further analysis (Edgar et al., 2011). The remaining high-quality sequences were clustered into operational taxonomic units (OTUs) at 97% identity using UPARSE v7.0.1090 algorithm (http://www.drive5.com/uparse/; Edgar, 2013). Taxonomic assignment of each OTU was determined using the RDP classifier v2.2 (Wang et al., 2007) with reference to Greengene gg_13_8 dataset. Metabolic characteristics of the microbiota were predicted according to the result of the 16S rRNA gene by PICRUSt v1.0.0 program (Langille et al., 2013) on Huttenhower Lab server (http://huttenhower.sph.harvard.edu/galaxy/root?tool_id=PICRUSt_normalize).

### Availability of data

The merged DNA sequence data were deposited in the genome sequence archive database (https://bigd.big.ac.cn) under the accession number CRA002095.

### Data analysis

The results for each parameter are presented as the mean ± standard error for each group. Principle coordinate analysis (PCoA) was conducted using QIIME pipeline 1.9.0. Non-parametric permutational multivariate analysis of variance (PERMANOVA) (Anderson, 2001) was applied to test the significance of differences between three or more groups using the R vegan package (Dixon, 2003). Principal components analysis (PCA) and canonical correspondence analysis (CCA) were also conducted using the R vegan package. The standard non-parametric Kruskal–Wallis test was used to detect the statistical significance of dominant OTUs (dOTUs), and the alpha-diversity indices of different sampling sites. Box plots were drawn using the ggpubr R package. Correlation analysis was also conducted using the R vegan package. Results with *P*-values of less than 0.05 were considered significantly significant.

## Results

**Figure 2:**
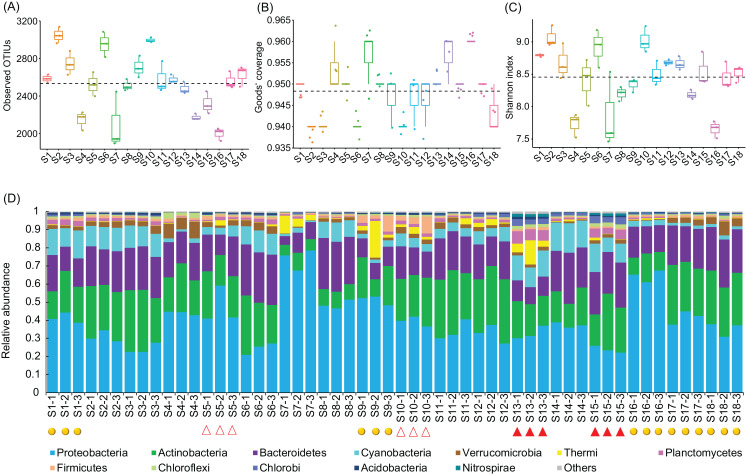
Observed OTUs (A), Goods’ coverage (B), Shannon index (C), and the dominant phyla (D) of each sampling site. The dominant phyla were defined by having a relative abundance of more than 1% in at least one sample. The red solid triangles at the bottom of the (D) profile indicate the samples collected from the sampling sites immediately downstream from the completed hydropower stations, the red hollow triangles indicate the samples collected from the sampling sites immediately downstream from the incomplete hydropower stations, and the yellow spheres indicate the samples collected from the sampling sites nearest to cities or towns.

A total of 2,565,459 (47,693.69 ± 1,934.22) high-quality sequences were obtained from the 54 samples. To eliminate the influence of sequencing depth, 24,848 high-quality sequences per sample were randomly resampled for further analysis. In total, 18,683 OTUs from 1,350 genera were identified at 97% sequence similarity. An average 2,534.78 ± 42.49 OTUs were detected from the sampling sites (Fig. 2A), which covered 94.79 ± 0.09% of all the predicted species (Fig. 2B). The Shannon indices of the water microbiota ranged from 7.46 to 9.26 (8.46 ± 0.06; Fig. 2C). There was not detected any significant difference in the number of observed OTUs (Kruskal–Wallis test, χ^2^ = 6.44, *p* = 0.09; Fig. 2A), the Goods’ coverage (Kruskal–Wallis test, χ^2^ = 2.11, *p* = 0.55; Fig. 2B), and the Shannon indices (Kruskal–Wallis test, χ^2^ = 6.96, *p* = 0.07; Fig. 2C) between different groups. These results implied that the construction of hydropower stations did not reduce the alpha diversity of the river water microbiota.

The OTUs belonged to 58 phyla (three Archaea phyla and 55 Bacteria phyla), with the exception of tiny unclassified sequences (0.106 ± 0.01%) (Table S1). However, consistent with previous studies (Wang et al., 2018), the microbiota were dominated by only 12 phyla, that is, Proteobacteria (40.42 ± 1.84%), Actinobacteria (22.13 ± 0.111%), Bacteroidetes (19.31 ± 0.81%), Cyanobacteria (7.37 ± 0.67%), Verrucomicrobia (2.59 ± 0.25%), Thermi (1.80 ± 0.48%), Planctomycetes (1.69 ± 0.21%), Firmicutes (1.46 ± 0.22%), Chloroflexi (0.92 ± 0.09%), Chlorobi (0.87 ± 0.14%), Acidobacteria (0.60 ± 0.05%), and Nitrospirae (0.24 ± 0.06%) (there relative abundances were more than 1% in at least one samples; Fig. 2D and Table S1), which was similar to results studied in other rivers (Kaevska et al., 2016). The relative abundances of the dominant phyla clearly fluctuated between the sampling sites (Fig. S2). In particular, the relative abundances of Acidobacteria, Chlorobi, Chloroflexi, Cyanobacteria, Nitrospirae, and Planctomycetes were significantly increased (Kruskal–Wallis test, *p* < 0.05) in the activated hydropower station group, while those of Proteobacteria was significantly decreased (Kruskal–Wallis test, *p* < 0.05; Fig. S3). Those results implied that the construction of hydropower stations obviously changed the water microbiota composition.

**Figure 3:**
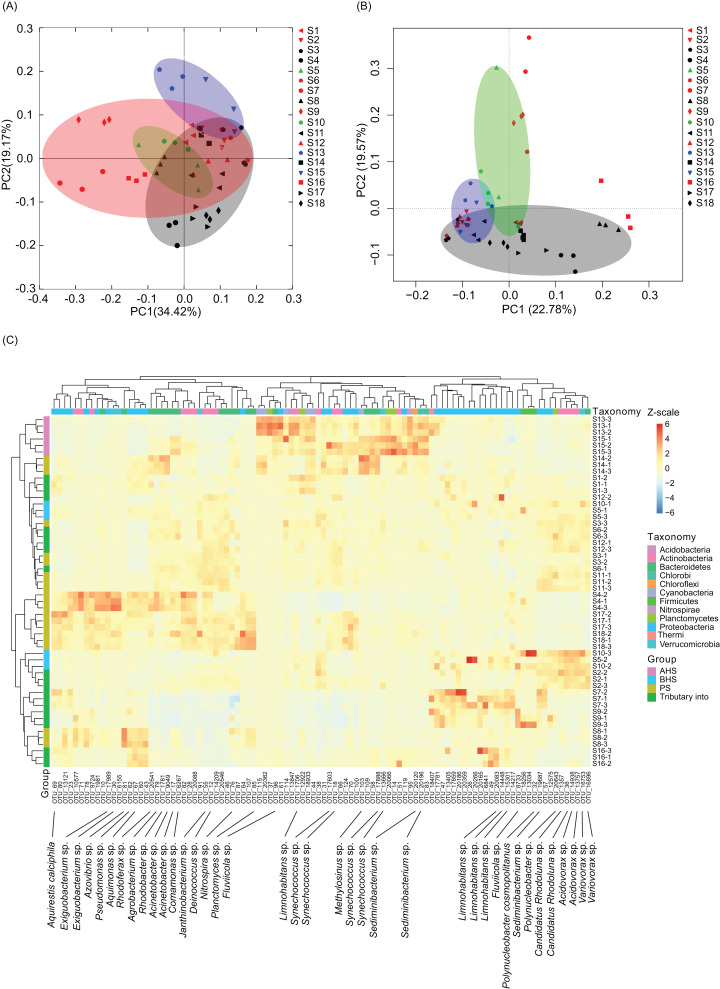
PCoA based on OTUs (A), PCA based on dominant OTUs (dOTUs, B), and the heatmap profile of top 100 significantly differential dOTUs (C) show differences of water microbiota between sampling sites. Dominant OTUs were those with a relative abundance of more than 0.1% in at least one sample (Huang et al., 2018). The sampling sites were divided into four groups according to the construction of the hydropower station and whether there is a tributary at the sampling site, that is, principal stream group (PS, black; S3, S4, S8, S11, S14, S17, and S18), tributary into group (red; S1, S2, S6, S7, S9, S12, and S16), building hydropower station group (BHS, green; S5 and S10), and activated hydropower station group (AHS, blue; S13 and S15) in the (A) and (B).

PCoA based on the weighted UniFrac distances between samples showed that the samples did not obviously cluster based on the river sections (Fig. 3A), nevertheless there was significant similarity between the three replicate samples at each sampling site (PERMANOVA, *F* = 4.06, *p* = 0.005). However, the microbiota of activated hydropower station group were significantly different with other samples (PERMANOVA, *F* = 4.11, *p* = 0.005; Fig. 3A). A total of 684 dominant OTUs (dOTUs, their relative abundance in at least one sample was more than 0.1%) were obtained from the 18,683 OTUs, all of which were bacteria (Table S2). The microbiota dOTUs compositions of activated hydropower station group were also significantly different with other samples (PERMANOVA, *F* = 4.44, *p* = 0.005; Fig. 3B). There were 315 dOTUs were detected significant difference between different groups (Kruskal–Wallis *H*-test, *p* < 0.05; Table S3). Cluster analysis based on the top 100 significantly differential dOTUs showed that the samples collected from the activated hydropower station group were clustered into one branch (Fig. 3C). Although the samples collected from the building hydropower station group were not significantly different from those from other sampling sites, *Synechococcus* dOTUs in Cyanobacteria were significantly enriched in the samples collected from the activated hydropower station group (Fig. 3C).

**Figure 4:**
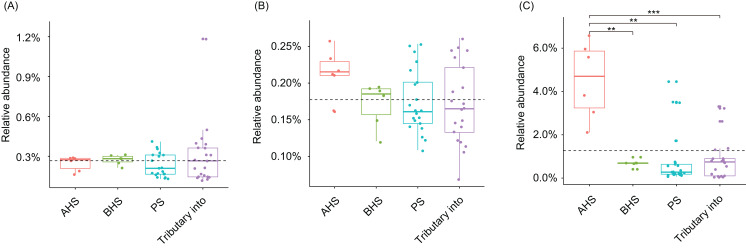
Relative abundances of *Phormidium* (A), *Planktothrix* (B), and *Synechococcus* (C), which were the major Cyanobacteria genera in the river water microbiota. AHS, activated hydropower station group; BHS, building hydropower station group; PS, principal stream group. **p < 0.01; ***p < 0.001.

*Cylindrospermopsis*, *Dolichospermum*, *Microcystis*, *Nostoc*, *Oscillatoria*, *Phormidium*, *Planktothrix*, *Gloeotrichia*, and *Synechococcus* were detected from the water samples, which were reported cyanobacterial harmful bloom genera (Paerl et al., 2016; Paerl, Otten & Kudela, 2018). However, their relative abundances were very low (Table 1). Only *Phormidium*, *Planktothrix*, and *Synechococcus* were detected from all of the sampling sites (Table 1). The relative abundance of *Synechococcus* in the activated hydropower station group were significantly higher than other groups (Kruskal–Wallis *H*-test, *p* < 0.01; Fig. 4).

**Table 1:** Relative abundance of cyanobacterial harmful bloom genera (mean ± S.E.).

Sampling site	Cylindrospermopsis	Dolichospermum	Microcystis	Nostoc	Oscillatoria	Phormidium	Planktothrix	Gloeotrichia	Synechococcus
S1	0.0013 ± 0.0013%	0	0.012 ± 0.0047%	0	0.0027 ± 0.0013%	0.35 ± 0.049%	0.20 ± 0.027%	0.0013 ± 0.0013%	3.1 ± 0.22%
S2	0.0013 ± 0.0013%	0.0027 ± 0.0013%	0.0040 ± 0%	0	0	0.69 ± 0.25%	0.22 ± 0.021%	0	0.85 ± 0.052%
S3	0	0.0013 ± 0.0013%	0.0040 ± 0.0023%	0.0013 ± 0.0013%	0.0027 ± 0.0027%	0.30 ± 0.017%	0.20 ± 0.027%	0	1.0 ± 0.361%
S4	0	0	0	0	0	0.14 ± 0.012%	0.13 ± 0.0048%	0	0.26 ± 0.048%
S5	0	0	0.0040 ± 0.0040%	0	0.0013 ± 0.0013%	0.26 ± 0.028%	0.15 ± 0.017%	0	0.68 ± 0.16%
S6	0	0.0013 ± 0.0013%	0.0054 ± 0.0036%	0	0	0.33 ± 0.017%	0.22 ± 0.016%	0.0013 ± 0.0013%	1.2 ± 0.17%
S7	0.0013 ± 0.0013%	0	0	0	0	0.14 ± 0.0094%	0.12 ± 0.0082%	0	0.075 ± 0.020%
S8	0	0.0054 ± 0.0036%	0.0013 ± 0.0013%	0	0	0.36 ± 0.031%	0.20 ± 0.017%	0	0.14 ± 0.039%
S9	0.0013 ± 0.0013%	0	0.0027 ± 0.0013%	0	0	0.22 ± 0.084%	0.11 ± 0.024%	0	0.050 ± 0.014%
S10	0.0013 ± 0.0013%	0	0.0013 ± 0.0013%	0	0	0.29 ± 0.0081%	0.19 ± 0.0013%	0	0.69 ± 0.0%
S11	0	0	0.0013 ± 0.0013%	0	0.0027 ± 0.0027%	0.19 ± 0.024%	0.16 ± 0.0088%	0	0.50 ± 0.11%
S12	0	0	0.0081 ± 0.0047%	0.0013 ± 0.0013%	0	0.23 ± 0.021%	0.19 ± 0.031%	0	0.59 ± 0.20%
S13	0	0	0	0	0.0027 ± 0.0013%	0.24 ± 0.038%	0.20 ± 0.021%	0	6.0 ± 0.29%
S14	0	0	0.0013 ± 0.0013%	0	0	0.33 ± 0.019%	0.24 ± 0.011%	0	3.8 ± 0.32%
S15	0	0	0	0	0.0027 ± 0.0013%	0.26 ± 0.033%	0.23 ± 0.014%	0	3.0 ± 0.50%
S16	0	0	0.0013 ± 0.0013%	0	0.0013 ± 0.0013%	0.14 ± 0.0013%	0.14 ± 0.015%	0.0013 ± 0.0013%	0.55 ± 0.090%
S17	0	0	0	0	0	0.16 ± 0.013%	0.14 ± 0.014%	0	0.20 ± 0.045%
S18	0	0	0.0054 ± 0.0036%	0	0	0.17 ± 0.0023%	0.15 ± 0.0067%	0	0.15 ± 0.0062%

**Figure 5:**
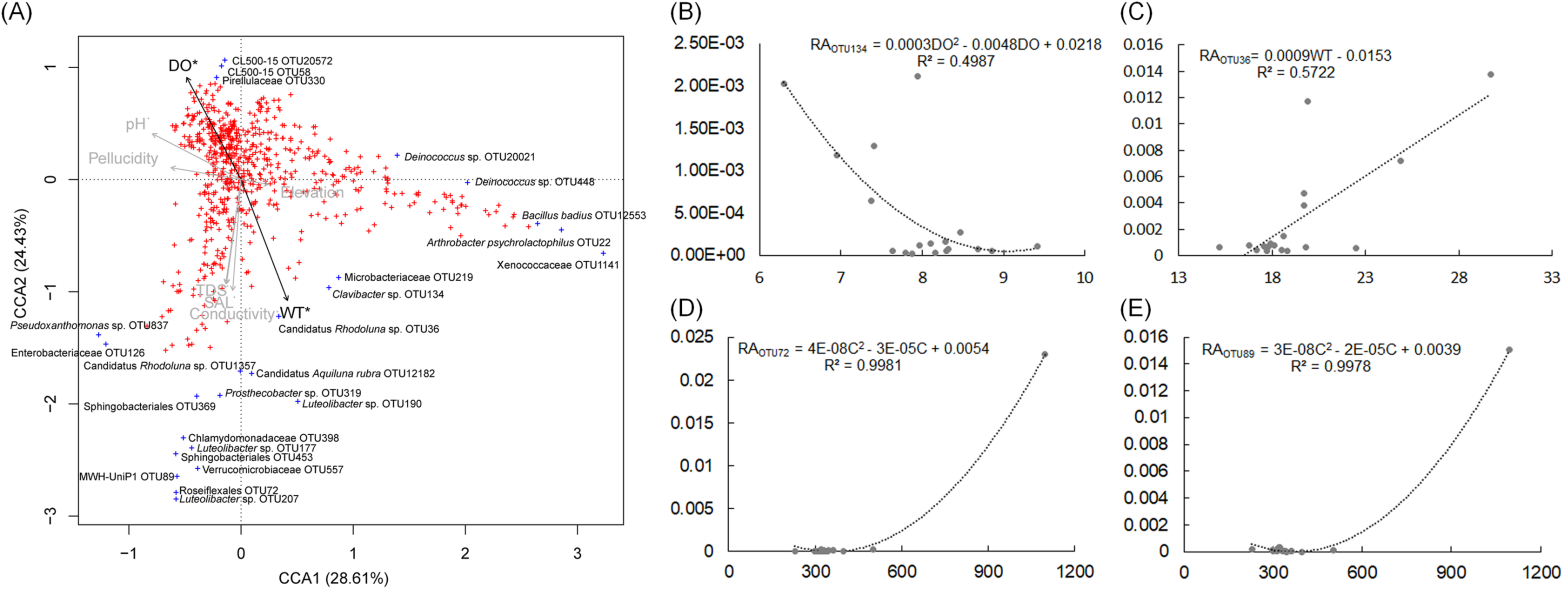
Canonical correspondence analysis profile based on dominant OTUs (A) and correlation analysis of the relative abundances of selected dominant OTUs and environmental factors (B–E) showing the correlation between dominant OTUs and environmental factors. RA, relative abundance; WT, water temperature; DO, dissolved oxygen; C, conductivity; TDS, total dissolved solids.

The pH and DO values of the sampling sites were stable, which were ranged from 7.5 to 8.32, and from 6.3 to 9.41, respectively (Table S4). The WT of sampling site S4 was evidently higher than other sampling sites (Table S4). CCA and regression analysis to analyze the impact of elevation, pH, DD, WT, conductivity, TDS, and salinity on the dOTUs. Our results showed that the measured environmental factors had a significant impact on the dOTUs (CCA with Monte Carlo permutation test, *F* = 1.82, *p* = 0.009). Further analysis showed that WT (Forward selection environmental variables, *r*^2^ = 0.60, *p* = 0.025) and DO (Forward selection environmental variables, *r*^2^ = 0.48, *p* = 0.020) significantly affected the dOTUs (Fig. 5A). Different environmental factors had impacts on different dOTUs. DO significantly negative impacted on OTU124 (*Clavibacter* sp.) (Fig. 5B), while WT significantly positive impacted on OTU36 (Candidatus *Rhodoluna* sp.) (Fig. 5C). Conductivity significantly positive impacted on OTU72 (Roseiflexales unidentified sp.) and OTU89 (MWH-UniP1 unidentified sp.) (Figs. 5D and 5E).

**Figure 6:**
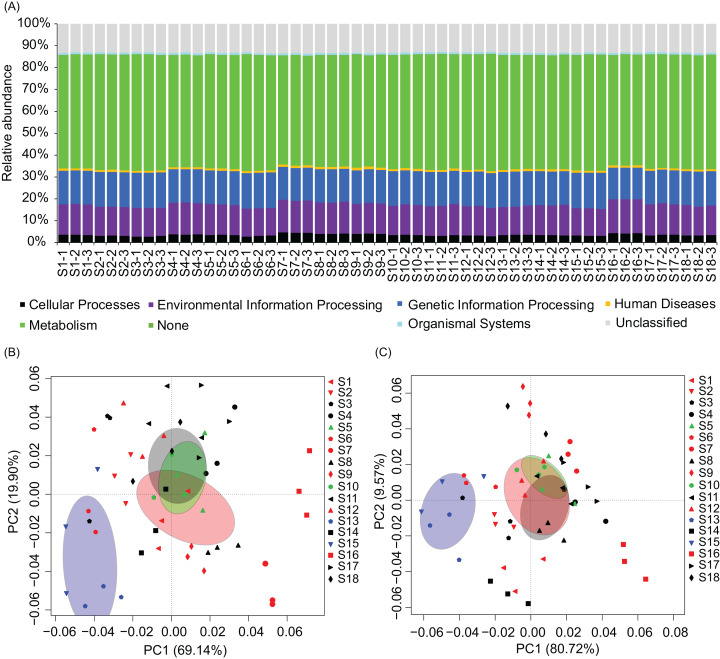
Metabolic characteristics of the river water microbiota predicted by the PICRUSt program. (A) Overall metabolic characteristics of all microbiota; (B) and (C), principal components analysis profiles based on all KEGG pathways and the significantly different KEGG pathways, respectively. The sampling sites were divided into four groups according to the construction of the hydropower station and whether there is a tributary at the sampling site, i.e. principal stream group (black; S3, S4, S8, S11, S14, S17, and S18), tributary into group (red; S1, S2, S6, S7, S9, S12, and S16), building hydropower station group (green; S5 and S10), and activated hydropower station group (blue; S13 and S15) in the (B) and (C). The ellipses indicate a 99% of confidence intervals.

To analyze metabolic characteristics of the microbiota, we predicted the metabolic characteristics of the microbiota by PICRUSt (Langille et al., 2013). About 80% of genes were involved in metabolism, genetic information processing, and environmental information processing (Fig. 6A). One hundred and thirty of the 328 KEGG pathways were significantly affected by the construction of hydropower stations (Fig. S4). PCA based on all KEGG pathways (PERMANOVA, *F* = 4.99, *p* = 0.005) and the significantly different KEGG pathways (PERMANOVA, *F* = 9.33, *p* = 0.005) showed the metabolic characteristics of water microbiota in the activated hydropower station group were significant difference to other groups (Figs. 6B and 6C). These results implied that the construction of the hydropower stations significantly changed the metabolic characteristics of the water microbiota.

## Discussion

Damming rivers and preexisting lakes have a global impact on natural water resources. Almost 800,000 artificial lakes and reservoirs have been built (Friedl & Wüest, 2002). Impoundments change the characteristics of a water body from “river” to “lake”, which affects not only their hydrology but also physical, chemical and biological characteristics, including increasing residence time, temperature, and stratification, and reducing turbulence (Friedl & Wüest, 2002). Although the influence of hydropower stations on the physical and chemical characteristics of river water has been widely studied (Wang et al., 2013; Chen et al., 2014; Fan, 2017), the effect of hydropower stations on the microbiota composition of river water has rarely been evaluated. In this study we evaluated the impact of hydropower station construction on the microbiota in the downstream of Jinsha River of China, covering sections upstream and downstream of two completed hydropower stations and two hydropower stations currently under construction, using high-throughput 16S rRNA gene sequencing. Our results showed that *Synechococcus* species were significantly enriched in the bacterial communities downstream of the complete hydropower stations. Given that *Synechococcus* species are reported that cause freshwater cyanobacteria algal blooms (Glibert et al., 2009; Wall et al., 2012; Nohomovich et al., 2013; Paerl et al., 2016), the increase in the proportion of *Synechococcus* increases the risk of freshwater algal blooms. It should be emphasized that although the sampling sites S13 and S15 were downstream of hydropower stations, they were very close to the stations (about 1 km), and given the water flow, the sampling sites likely represent the situation upstream. Because hydropower stations can significantly reduce upstream water flow, which is more likely to cause algal blooms (Paerl & Huisman, 2008; Chung, Lee & Jung, 2008; Hudnell et al., 2010; Xiong et al., 2013; Paerl et al., 2016; Paerl, Otten & Kudela, 2018; Ji et al., 2017; Bae & Seo, 2018), our results implied that hydropower stations increase the risk of upstream freshwater algal blooms.

The construction of hydropower stations changes the physical and chemical factors of river water, such as WT and DO (Chen, Deng & Liang, 2010), which significantly influence aquatic microbiota composition (Yan et al., 2017; Wang et al., 2018; Ni et al., 2018). In this study, our results showed that WT and DO significantly influenced the dOTUs composition of water microbiota (Fig. 5A). WT was also a major factor affecting phytoplankton growth (Butterwick, Heaney & Talling, 2005; Paerl et al., 2016). Our results showed that the proportion of Cyanobacteria were significantly increased in the activated hydropower station group, which would increase the primary productivity and the content of algal toxins in the water. Considering algal toxins significantly changes the intestinal flora of animals on the higher food chain in water (Macke et al., 2017; Duperron et al., 2019), and the intestinal microbiota plays an important role in multiple physiological functions of aquatic animals (Pérez et al., 2010; Ni et al., 2014), the algal toxins probably affect the health of organisms on the higher food chain of the whole water body. However, the specific impact needs to be further investigated and evaluated. In addition, maximal growth rates of most harmful algal bloom caused Cyanobacteria occur at more than 25 °C (Butterwick, Heaney & Talling, 2005; Paerl et al., 2016). However, the WTs of the sampling sites were less than 25 °C with the exception of the S4. This may be one of the main reasons for the low relative abundance of the cyanobacterial harmful bloom genera (Table 1). This also means that the risk of cyanobacterial harmful bloom caused by the construction of hydropower stations in summer may be higher when the WT is generally over 25 °C.

The samples were clustered into two groups according to the cluster analysis based on the top 100 significantly differential dOTUs (Fig. 3C). However, we did not find out which factor caused the difference. In many cases, three repeated samples from the same sampling site were clustered together (Fig. 3C), which indicated that the specific characteristics of the sampling sites casted specific microbiota compositions in the river water. Although the sampling sites were connected (Fig. 1), our results did not show the impact of the upstream microbiota on the downstream microbiota. Considering that there were many tributaries flowing into the sampling river section (Fig. 1), it was possible to change the nutrient compositions and other physical and chemical factors of the water at different points, which formed the unique feature and microbiota compositions of each sampling site.

PICRUSt program is widely used to predict the metabolic characteristics of microbiota (such as Li et al., 2018; Ni et al., 2018). Our results showed that the construction of the hydropower stations significantly changed the metabolic characteristics of the water microbiota. Considering the changes in the predicted metabolic characteristics at the DNA level, these results implied that the bacterial species with different metabolic characteristics in the water microbiota were replaced after the construction of the hydropower station.

## Conclusions

Our results showed that hydropower stations significantly increased the relative abundance of Cyanobacteria in the microbiota of the water and thus increased the risk of algal blooms. However, the mechanism causing the increase of the relative abundance of Cyanobacteria and how to prevent the risk of algal blooms caused by hydropower stations should be further studied.

## Supplemental Information

10.7717/peerj.9500/supp-1 Supplemental Information 1 Phylum compositions of the water microbiota. Bold font indicates the dominant phyla (there relative abundances were more than 1% in at least one samples).

10.7717/peerj.9500/supp-2 Supplemental Information 2 Dominant OTUs detected from the river water microbiota.

10.7717/peerj.9500/supp-3 Supplemental Information 3 Significantly different dOTUs between the different groups.

10.7717/peerj.9500/supp-4 Supplemental Information 4 Environmental factors and grouping of the sampling sites. WT, water temperature; DO, dissolved oxygen; TDS, total dissolved solids.

10.7717/peerj.9500/supp-5 Supplemental Information 5 Pictures of sampling sites. (A)-(R), sampling sites from S1 to S18.

10.7717/peerj.9500/supp-6 Supplemental Information 6 Relative abundances of dominant phyla from each sampling site.

10.7717/peerj.9500/supp-7 Supplemental Information 7 Relative abundances of significantly different dominant phyla from each group. The sampling sites were divided into four groups according to the construction of hydropower station and whether there is a tributary at the sampling site, i.e. principal stream group (S3, S4, S8, S11, S14, S17, and S18), tributary into group (S1, S2, S6, S7, S9, S12, and S16), building hydropower station group (S5 and S10), and activated hydropower station group (S13 and S15). (A), Acidobacteria; (B), Bacteroidetes; (C), Chlorobi; (D), Chloroflexi; (E), Cyanobacteria; (F), Firmicutes; (G), Nitrospirae; (H), Planctomycetes; (I), Proteobacteria; (J), Thermi; (K), Verrucomicrobia. The significant difference was detected using Kruskal-Wallis test with wilcox.test by R ggpubr package. *, p < 0.05; **, p < 0.01; ***, P < 0.001.

10.7717/peerj.9500/supp-8 Supplemental Information 8 Heatmap showing the relative abundance of KEGG pathways of the water microbiota. The data were transformed to Z-scale by R pheatmap package.

## Additional Information and Declarations

### Data Availability

The following information was supplied regarding data availability: The DNA sequence data are available in BIGD: CRA002095. https://bigd.big.ac.cn/bioproject/browse/PRJCA001909.
