# Supplementary figures and images for: Influence of hydropower stations on the water microbiota in the downstream of Jinsha River, China
Source: PeerJ. 2020 Jul 16;8:e9500. doi: 10.7717/peerj.9500 (PMC7369022; doi:10.7717/peerj.9500)

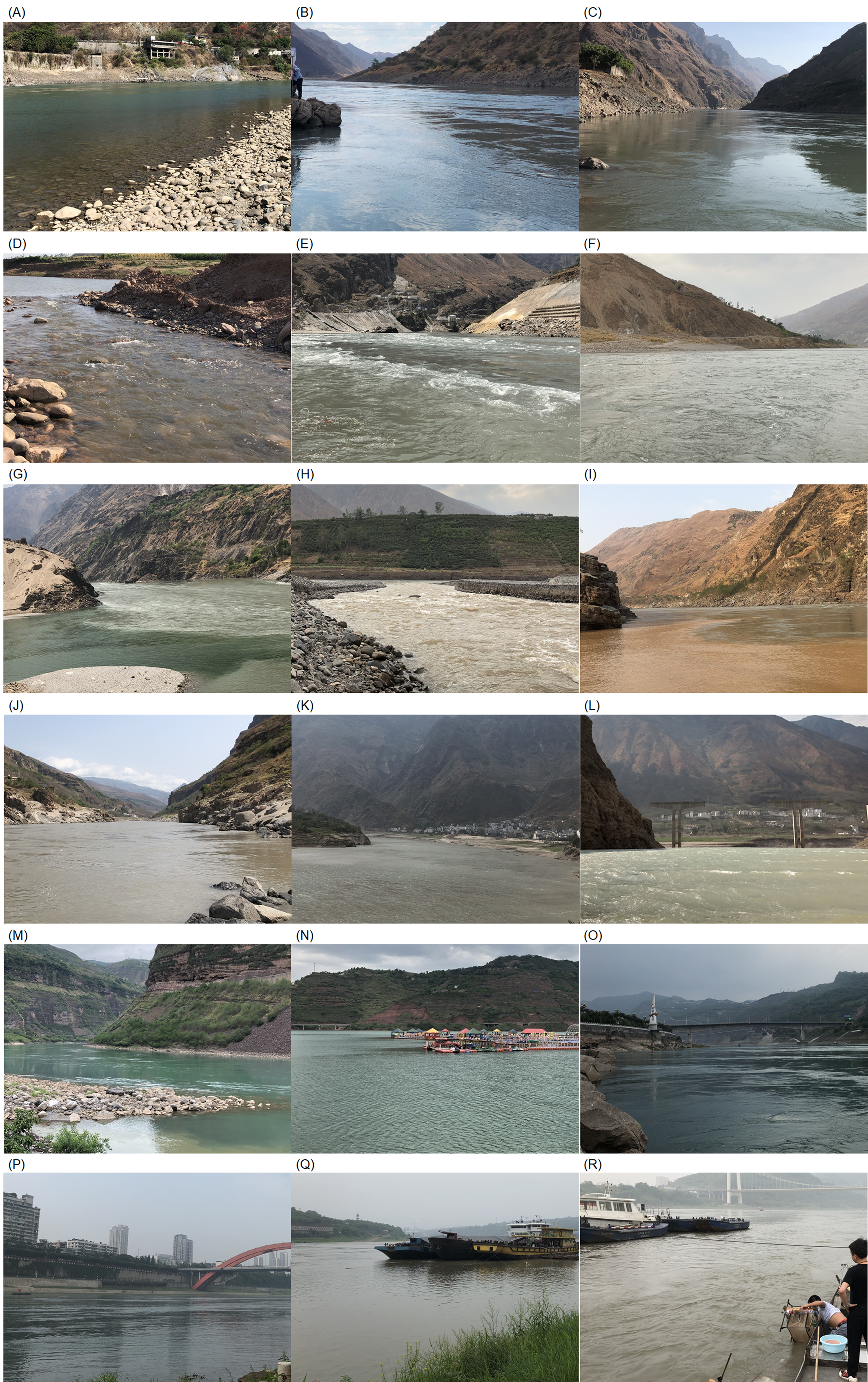

Supplement: Supplemental Information 5 [file peerj-08-9500-s005.png]

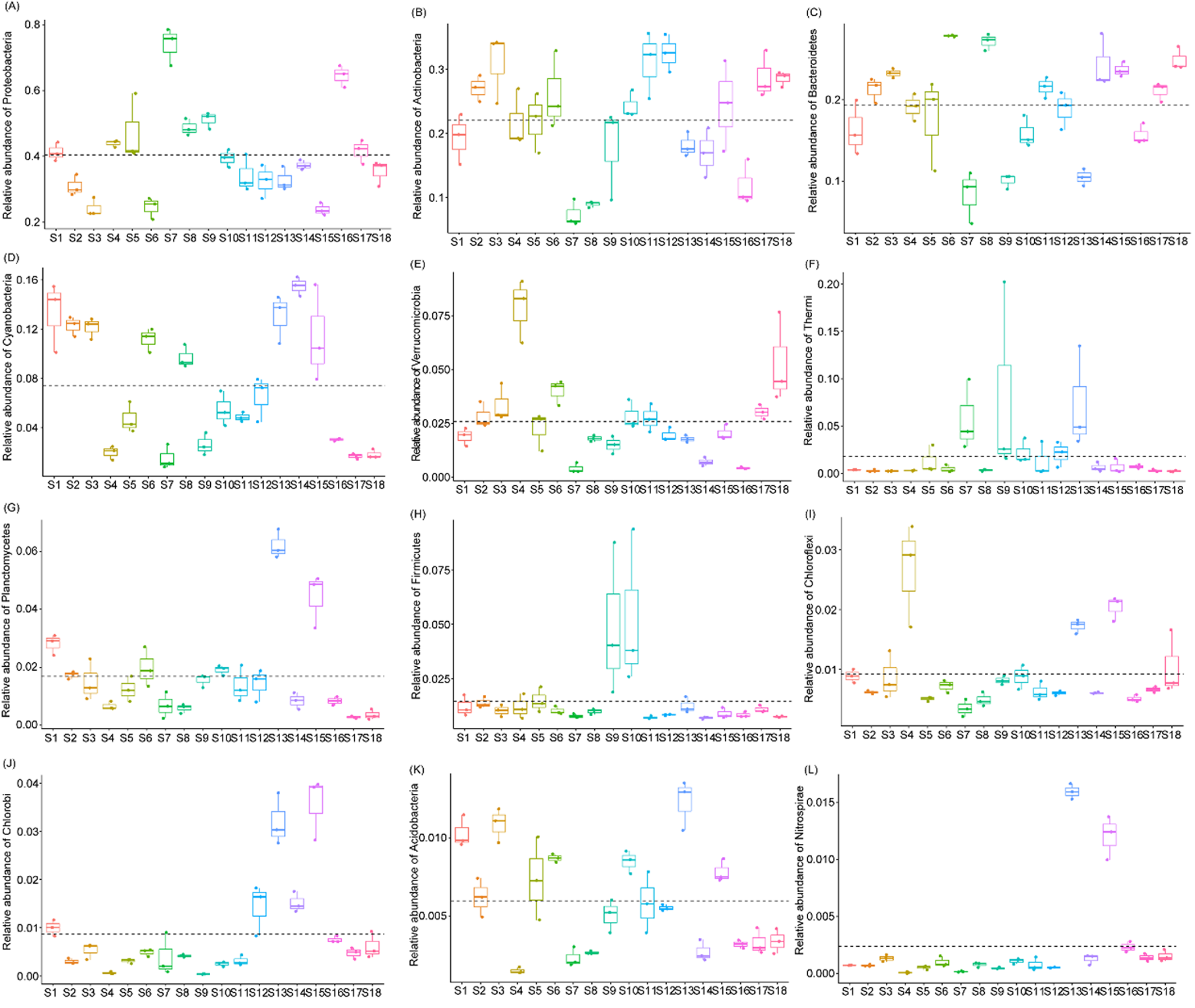

Supplement: Supplemental Information 6 [file peerj-08-9500-s006.png]

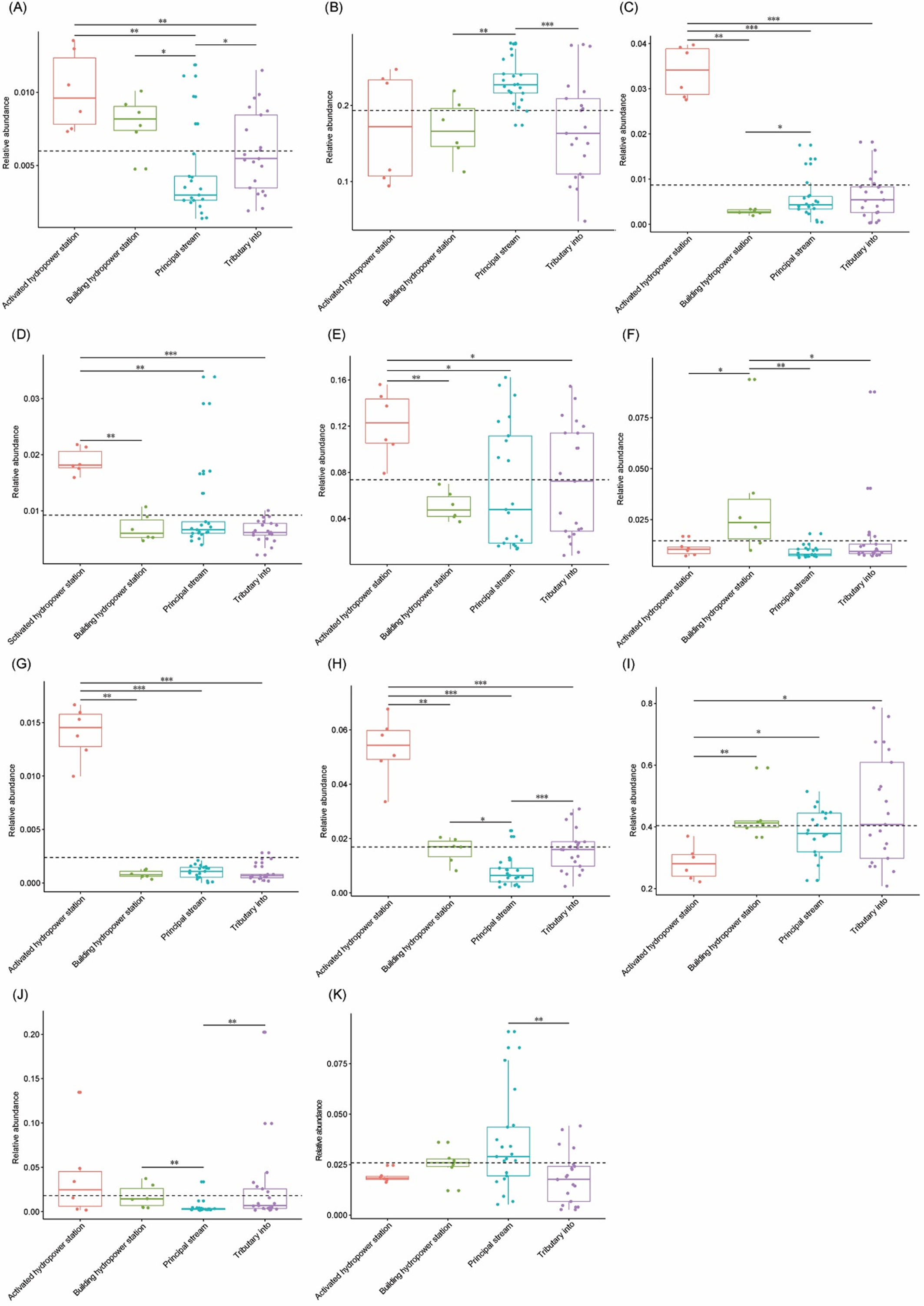

Supplement: Supplemental Information 7 — The sampling sites were divided into four groups according to the construction of hydropower station and whether there is a tributary at the sampling site, i.e. principal stream group (S3, S4, S8, S11, S14, S17, and S18), tributary into group (S1, S2, S6, S7, S9, S12, and S16), building hydropower station group (S5 and S10), and activated hydropower station group (S13 and S15). (A), Acidobacteria; (B), Bacteroidetes; (C), Chlorobi; (D), Chloroflexi; (E), Cyanobacteria; (F), Firmicutes; (G), Nitrospirae; (H), Planctomycetes; (I), Proteobacteria; (J), Thermi; (K), Verrucomicrobia. The significant difference was detected using Kruskal-Wallis test with wilcox.test by R ggpubr package. *, p < 0.05; **, p < 0.01; ***, P < 0.001. [file peerj-08-9500-s007.png]

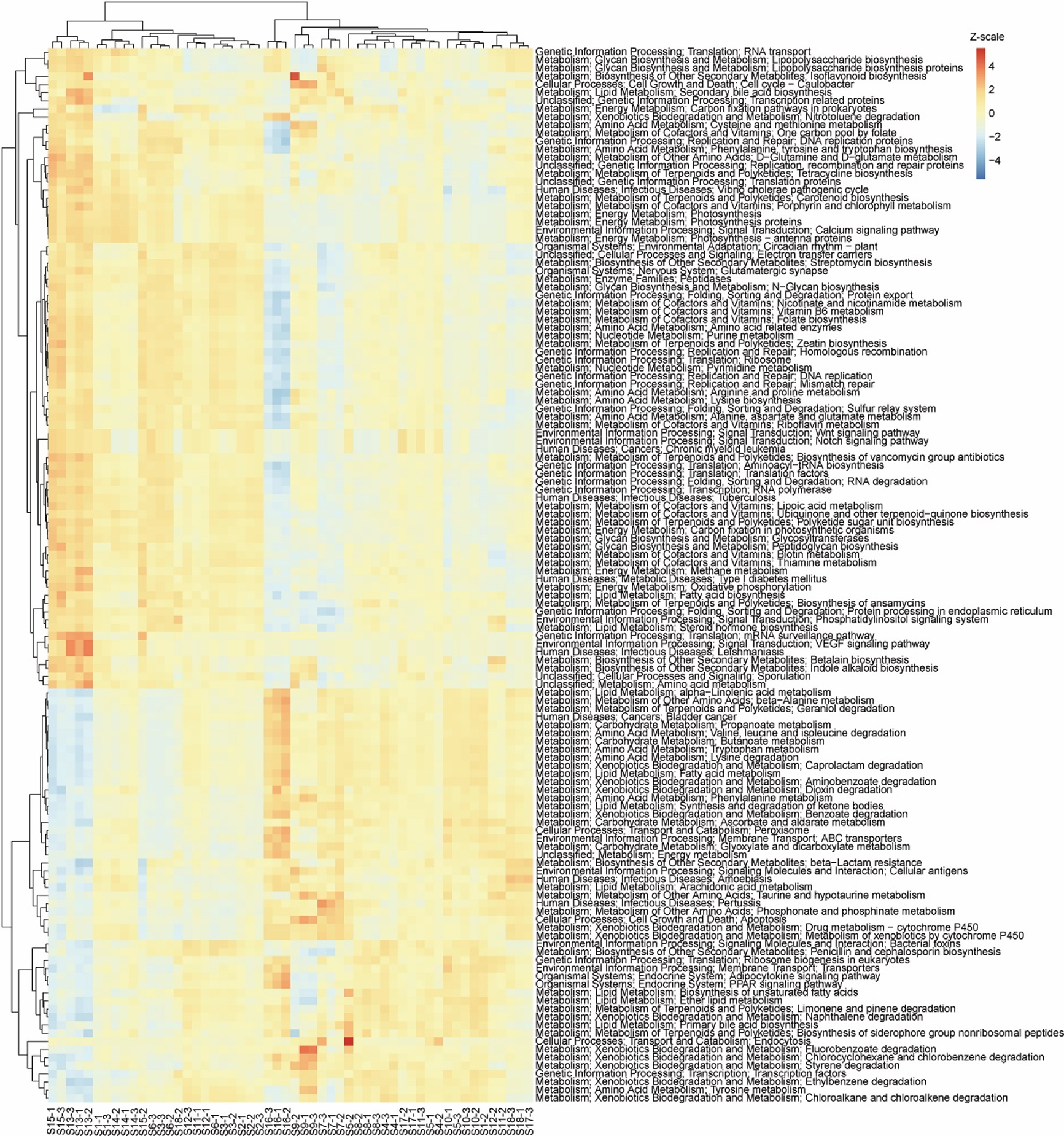

Supplement: Supplemental Information 8 — The data were transformed to Z-scale by R pheatmap package. [file peerj-08-9500-s008.png]
